# Supplementary material for: Cholesterol promotes the formation of dimers and oligomers of the receptor tyrosine kinase ROR1
Source: J Biol Chem. 2025 Dec 3;302(1):111000. doi: 10.1016/j.jbc.2025.111000 (PMC12800696; doi:10.1016/j.jbc.2025.111000)
Supplement: Supplementary_Information [file mmc1.docx]

**Cholesterol promotes the formation of dimers and oligomers of the receptor tyrosine kinase ROR1**

Alyssa Ward^1^, Luis J. Baeza-Ballesteros^2^, Ryan Schuck^1^, Maria J. García-Murria^2^, Rajan Lamichhane^1^, Ismael Mingarro^2^, and Francisco N. Barrera^1, *^

^1^Department of Biochemistry & Cellular and Molecular Biology, University of Tennessee, Knoxville, USA

^2^Departament de Bioquímica i Biologia Molecular, Institut Universitari en Biotecnologia i Biomedicina (BioTecMed), Facultat de Ciències Biològiques, Universitat de València, E-46100 Burjassot, Spain.

^*^Correspondence: fbarrera@utk.edu

**Supplementary Information for Ward *et al*. contains:**

**Supplementary Figures S1-S12.**

**Supplementary Tables S1 & S2**

**
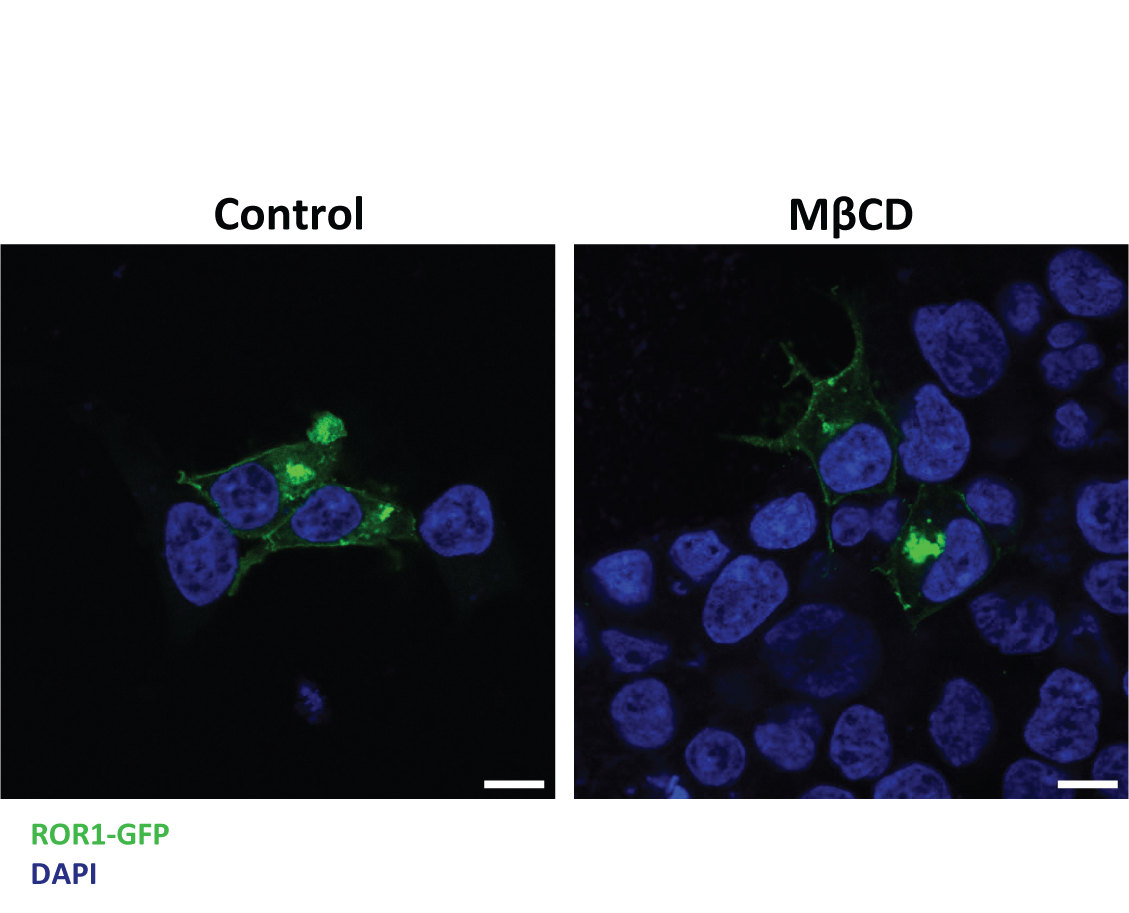
**

**Figure S1: Confocal microscopy of ROR1-GFP expressed in HEK293T cells.** Expression of ROR1-GFP under control (*left*) and MβCD (*right*) conditions. Cell nuclei were stained with DAPI. Scale bars represent 10 µm.


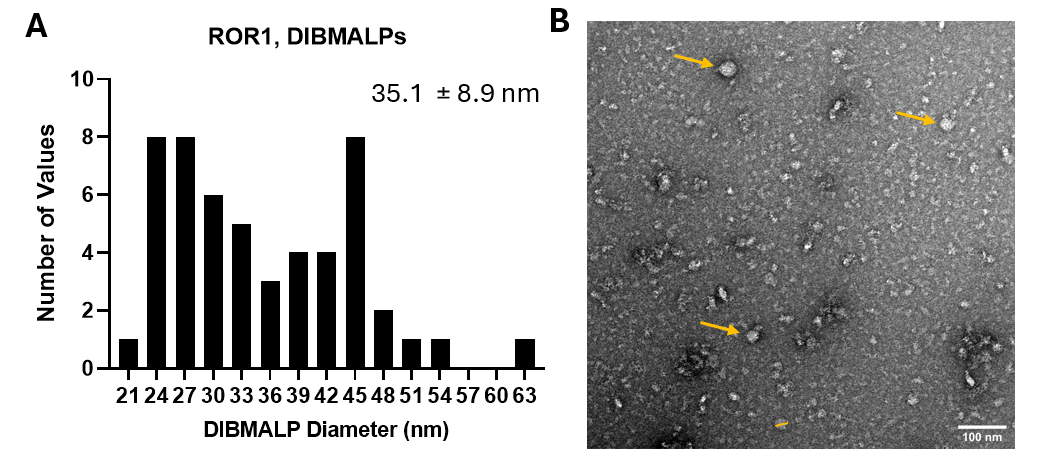

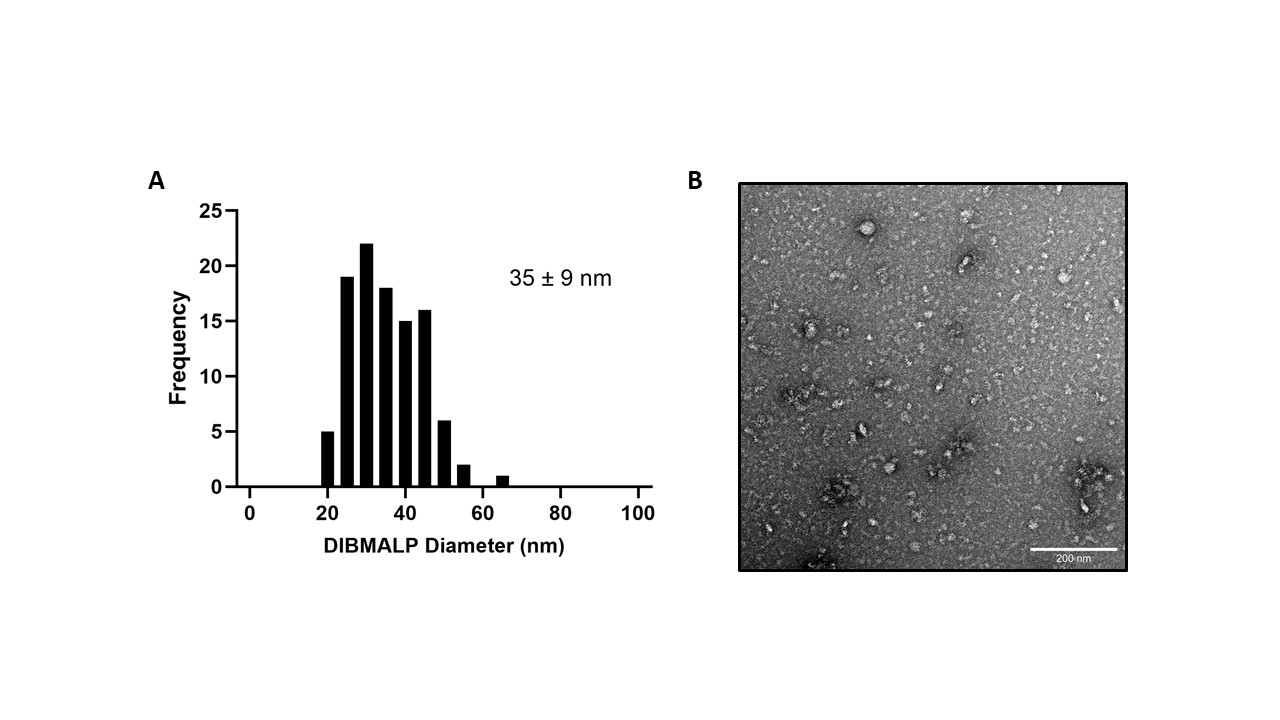


**Figure S2: Quantification of DIBMALP diameter by transmission electron microscopy (TEM).** DIBMALPs prepared from isolated membrane fractions from HEK293T cells that were transfected with ROR1-GFP were negative stained and imaged by TEM. Nanodisc diameter was quantified to be 35 ± 9 nm (mean ± S.D.). **(A)** Population distribution of DIBMALPs. **(B)** Representative TEM image of DIBMALPs. Images were collected and quantified from three biological replicates. Yellow arrows indicate individual DIBMALPs with the yellow line representing an example of the DIBMALP diameter measurement.


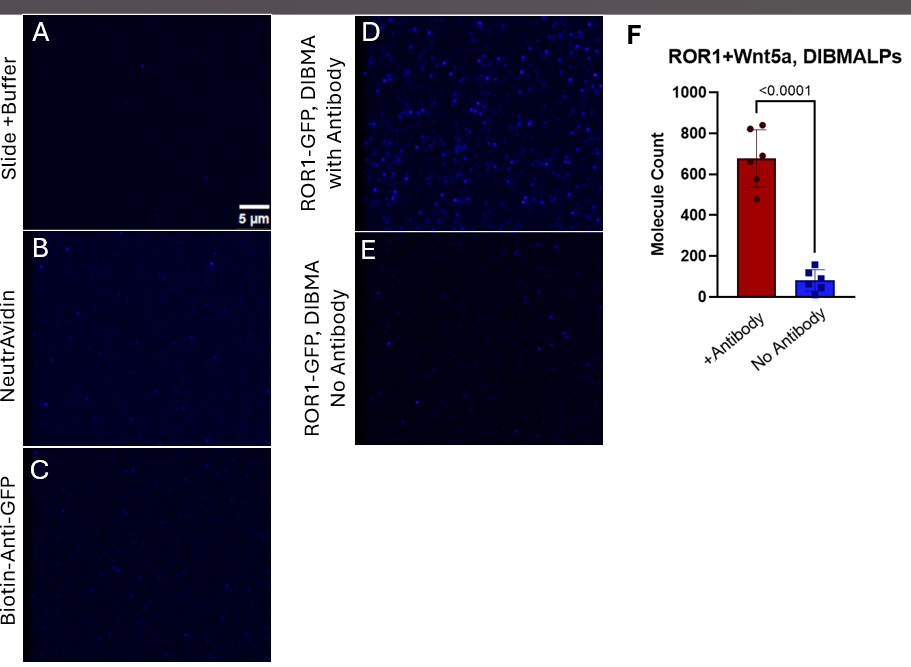


**Figure S3: Controls of single molecule pulldown TIRF of ROR1-GFP.** **(A-C)** Representative TIRF images of a prepared slide after rinse with buffer, addition of NeutrAvidin, and incubation with the biotinylated GFP antibody. **(D-F)** Quantification of the number of molecules counted per 6 recorded TIRF movies of slides incubated with DIBMALPs prepared from ROR1-GFP transfected HEK293T cell membrane fractions, treated with Wnt5a, and after incubation with or without the biotinylated GFP antibody on the slide (representative images are shown in **(D, E)**). An unpaired *t*-tests was run for statistical analysis.

**Figure S4: Stimulation by Wnt5a or Wnt3a does not affect ROR1 self-assembly.** Percent of 1 step, 2 step, and ≥3 step populations of ROR1 isolated in DIBMALPs prepared from HEK293T cells and treated with Wnt5a (0.5 µg/mL) or Wnt3a (0.2 µg/mL). A two-way ANOVA followed by a multiple comparison unpaired *t*-tests was run for statistical analysis and showed no significance, N=3 (each represented by a solid dot).


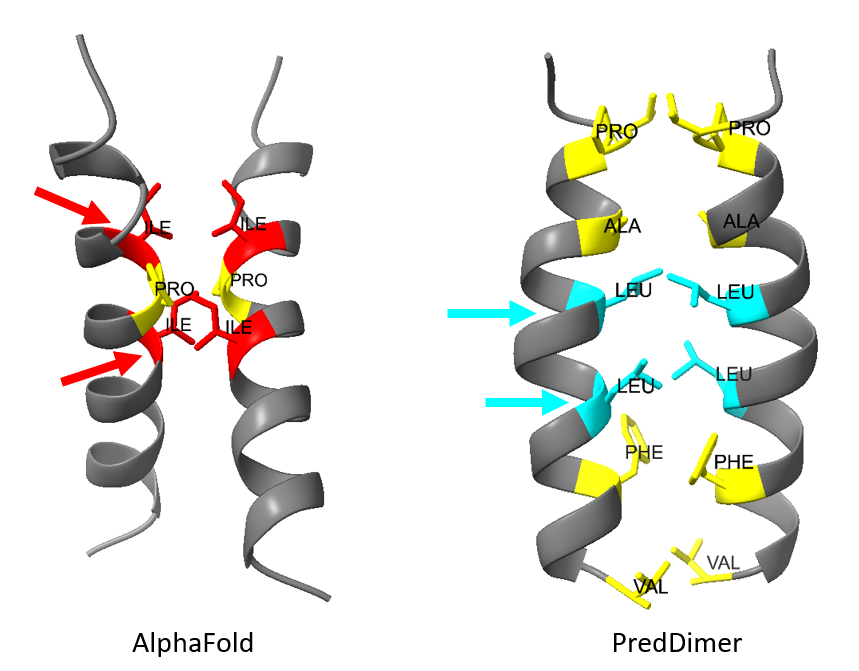


**Figure S5: ROR1 TMD Predicted Dimerization Interfaces.** *(Left)* AlphaFold predicted contact sites between ROR1 TMD helices. Highlighted residues represent predicted contact sites within 3 Å. Residues highlighted in red (Ile 414 and Ile 418) were mutated in Mut 1. *(Right)* Dimerization interface predicted by PredDimer (F-Score: 2.334). Residues represented in cyan (Leu 416 and Leu 420) were mutated in Mut 2. Residues highlighted in yellow represent additional residues predicted to form contacts or located at the TMD dimer interface. Arrows indicate location of Mut 1 and Mut 2 amino acid-pairs substitution locations. Sequence used for bioinformatic analysis: ILVPSVAIPLAIALLFFFICV.

**Figure S6: Mutation of dimerization interfaces of the ROR1 TMD in HEK293T cells. (A)** Amino acid sequences of wild-type and mutant ROR1 TMDs fused to VFP halves for the BiFC assay and their predicted Δ*G* (Δ*G*_app_) values for membrane insertion in kcal/mol. **(B)** Relative fluorescence units (RFU) of VFP fused TMD ROR1 wild-type and mutants. Mean ± S.D. are shown with each individual experiment represented by a dot. **(C)** Helical wheel projection of ROR1 TM helix highlighting in red and blue the mutated Ile- and Leu-pairs, respectively.


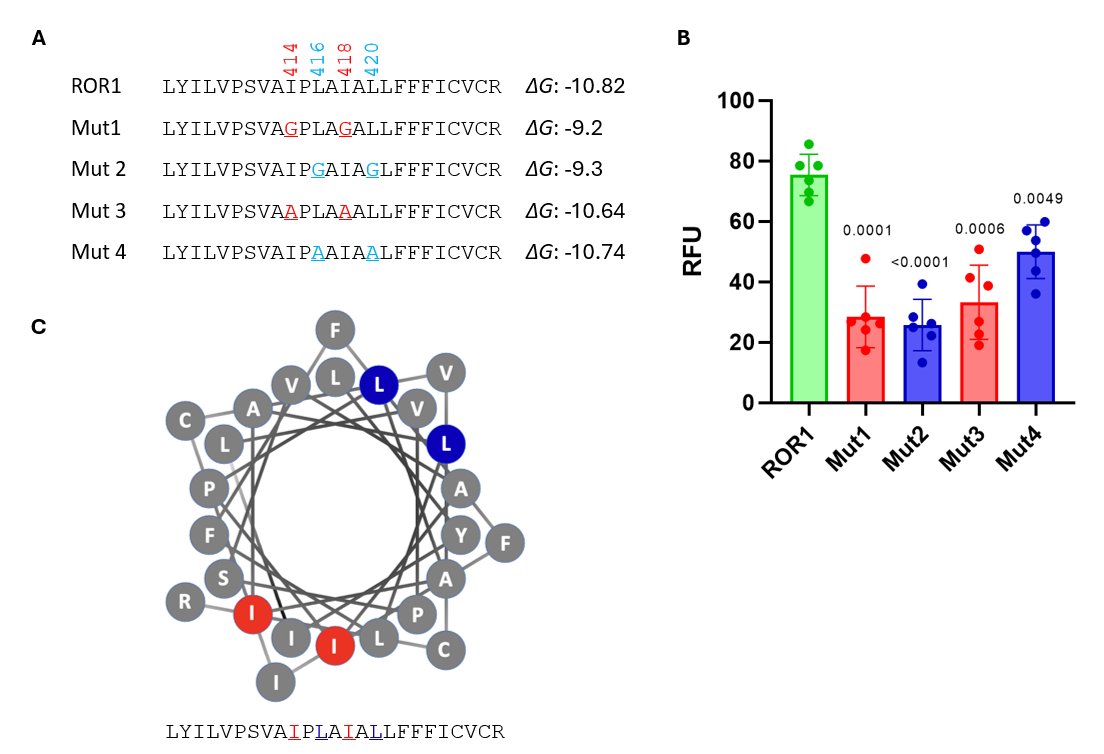

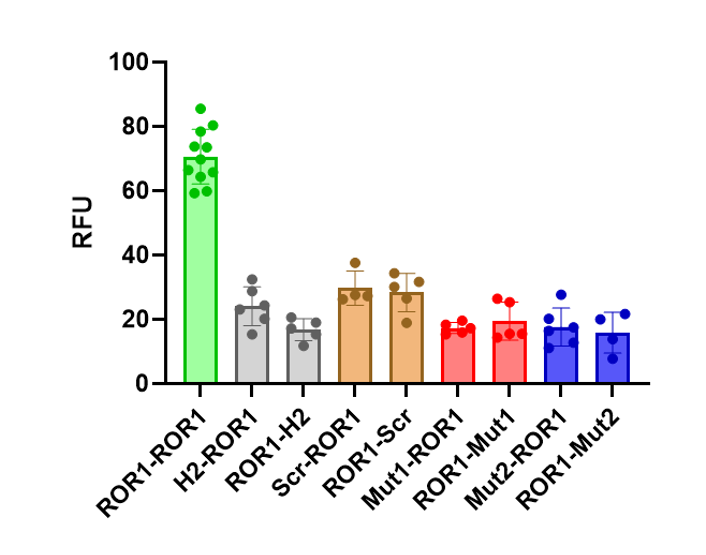


**Figure S7:  Measurement of the interaction between ROR1 and the rest of the TMDs.** Relative fluorescence units (RFU) of each homo-oligomer tested in the BiFC assay. ROR1 homo-oligomerizes, as it shows levels significantly higher than the other homo-oligomers (two-tailed homoscedastic *t*-test) is highlighted in green. The rest of heterodimers are colored with different colors for each pair of TM segments in both combinations. The mean ± S.D. of at least 4 independent experiments are shown. The individual value of each experiment is represented by a solid dot.


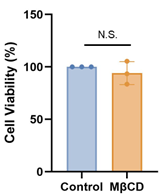


**Figure S8: Cholesterol removal does not affect cell viability.** MTS assay quantification of HEK293T cells treated with and without MβCD. An unpaired *t*-test was used for statistical analysis. N=3 (each represented by a solid dot).

**
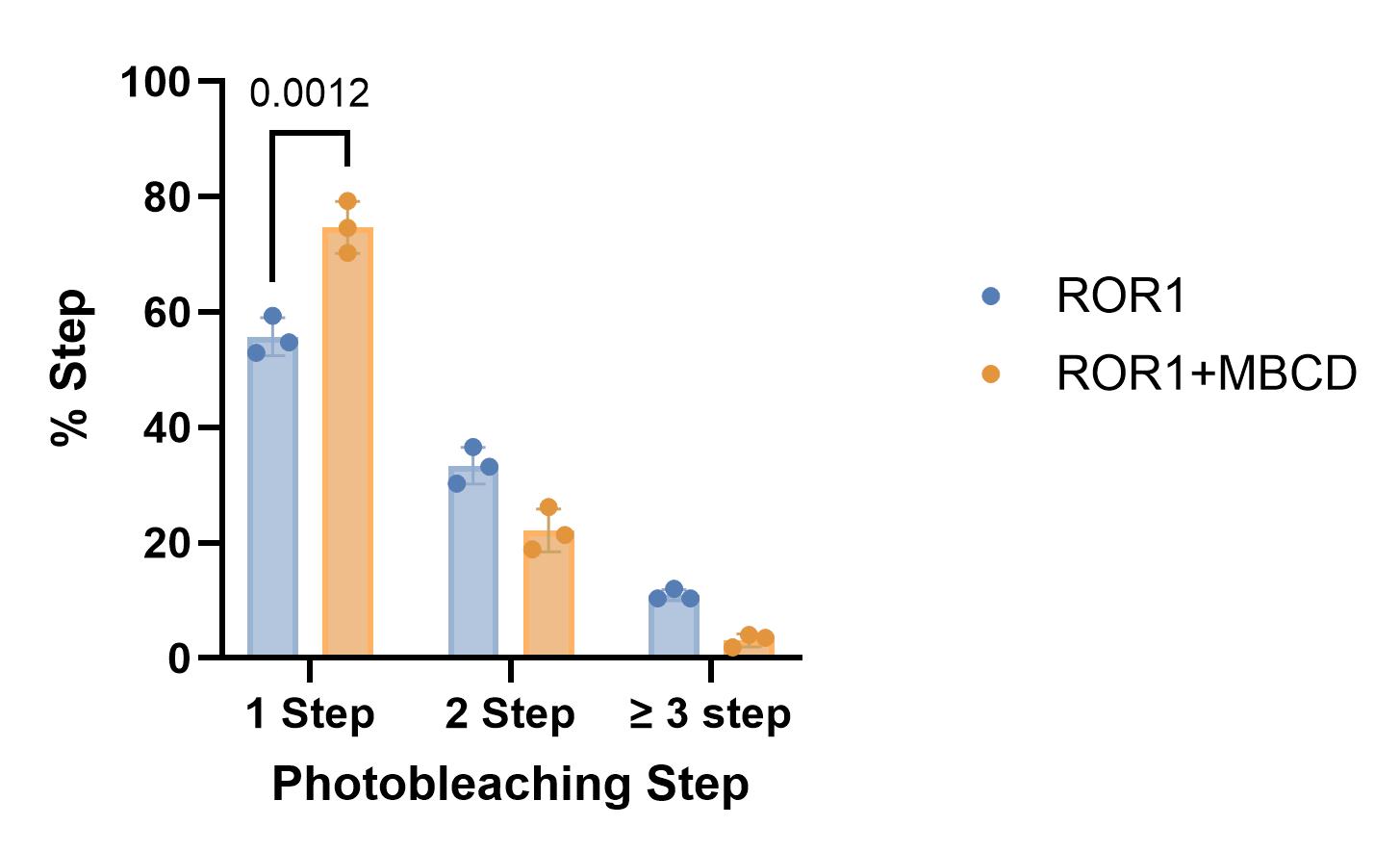
**

**Figure S9: Membrane Chol promotes ROR1 self-assembly.** Percent step analysis of SiMPull-POP results for ROR1 isolated in DIBMALPs from HEK293T cells in control and MβCD treated conditions. A two-way ANOVA followed by a multiple comparison unpaired *t*-tests was run for statistical analysis. N=3 (each represented by a solid dot).

**
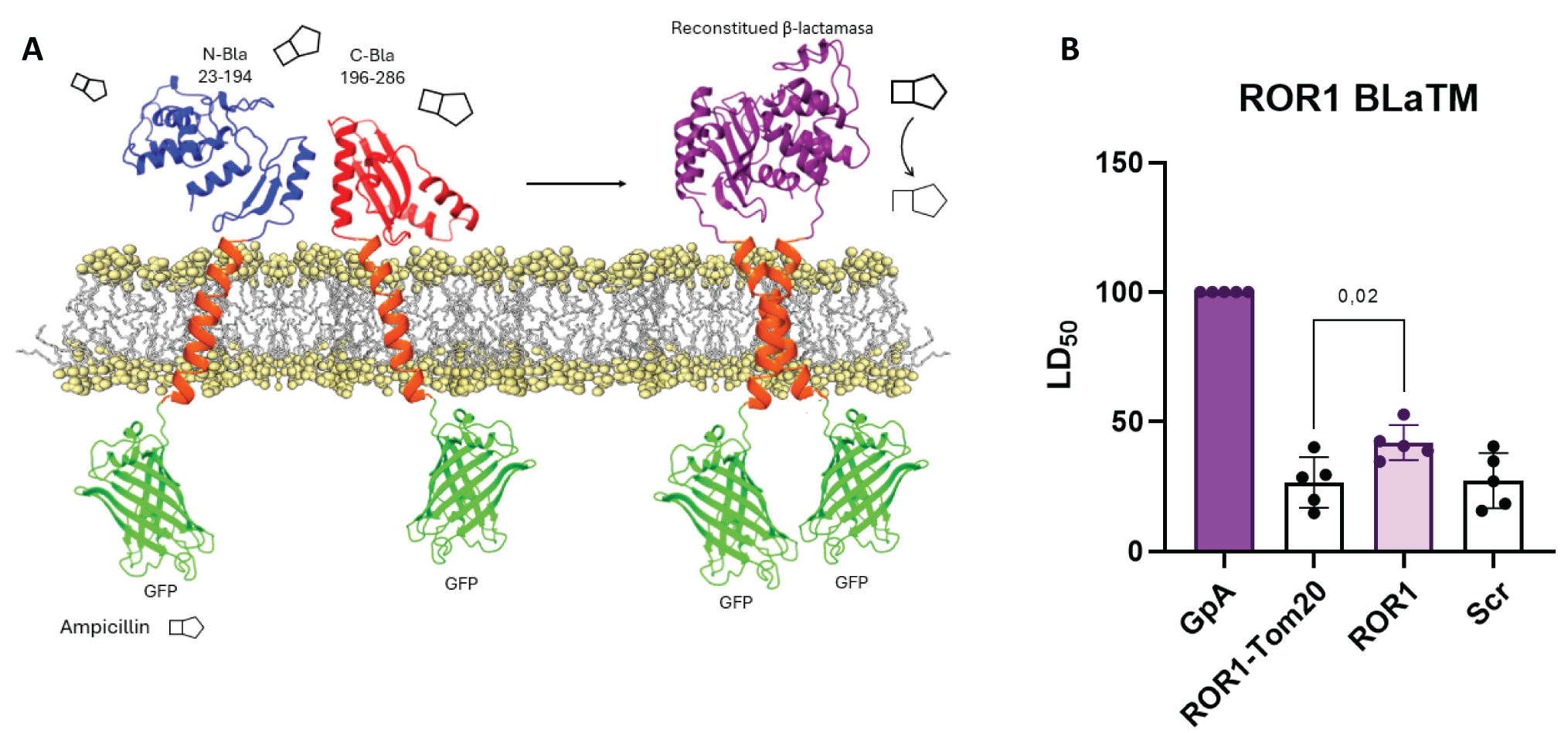
**

**Figure S10: ROR1 TMD interactions in bacterial membranes**. **(A)** Schematic representation of the β-Lactamase complementation (BLaTM) assay. **(B)** The βN and βC chimeras bearing the TMD of the indicated proteins were co-expressed in *E. coli*, and the resulting ampicillin LD_50_ was measured. The βN ROR1–βC T20 heterodimer was used as a negative control (white), and the βN GpA–βC GpA homodimer was used as a positive control (purple) and normalization value across experimental replicates. The normalized means ± S.D. of at least three independent experiments (n ≥ 5) are shown. The individual value for each experiment is represented by a solid dot. An interaction was considered positive if the observed LD_50_ was significantly higher (two-tailed homoscedastic *t*-test, P value < 0.05) than the negative control.


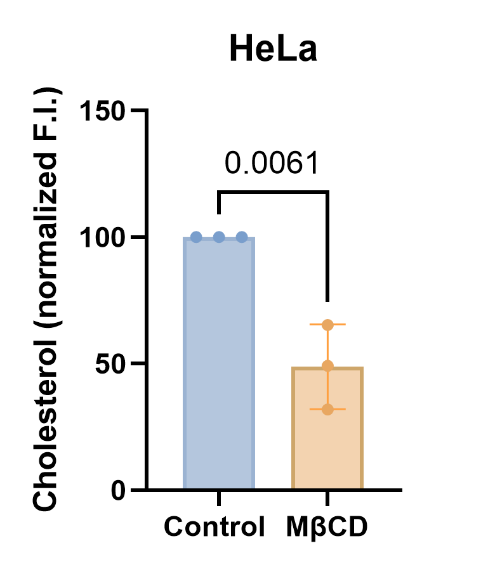


**Figure S11: Quantification of the removal of membrane Chol from HeLa cells.** Quantification of **(A)** cell viability (MTS assay) and **(B)** cholesterol levels of HeLa cells in control and MβCD treated conditions. An unpaired *t*-tests was run for statistical analysis for both assays. N=3 (each represented by a solid dot).


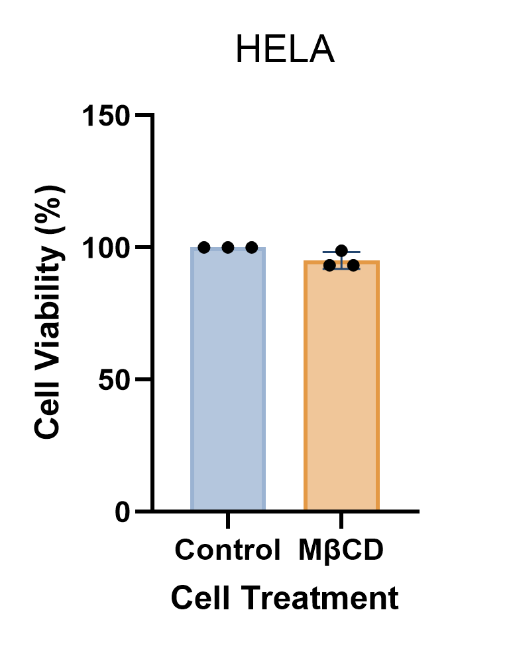


**A**

**B**

**Figure S12:** Plasmid map of the pCAG-ROR1-GFP construct, annotated by GENEWIZ and viewed in the SnapGene software.


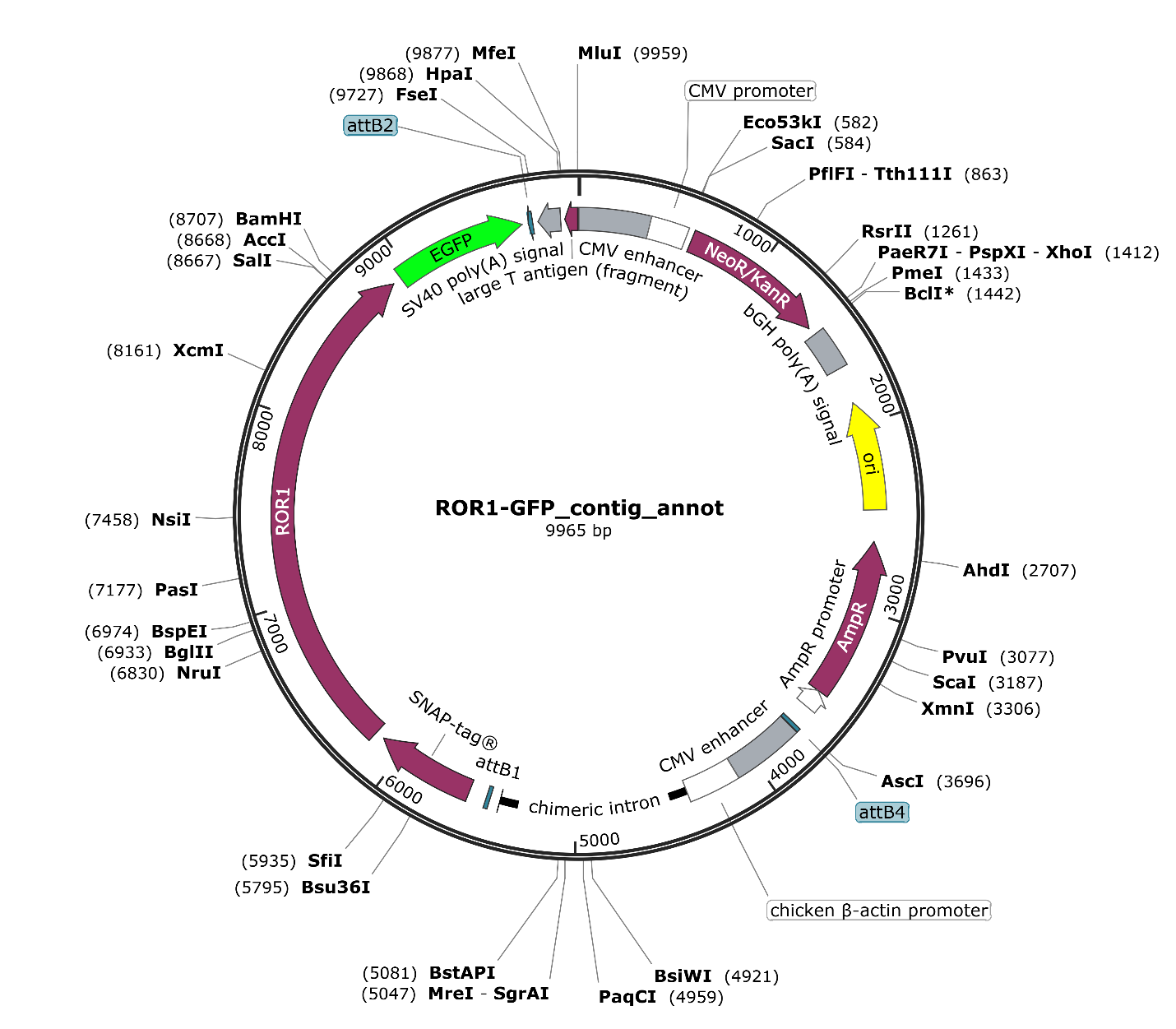


**Table S1:** **Annotated amino acid sequence of the pCAG-ROR1-GFP construct.** Plasmid DNA was sequenced using Next Generation Sequencing by GENEWIZ (Plasmid-EZ). Files were annotated and translated into the amino acid sequence in SnapGene. The construct begins with the ROR1 target sequence followed by a SNAP-tag, an N-terminal linker, the ROR1 protein sequence, and a C-terminal linker that precedes an EGFP and x10 His-tag (individual construct components are separated by []).

| **ROR1-GFP construct amino acid sequence** |
| --- |
| [MHRPRRRGTRPPLLALLAALLLAARGAAA][DKDCEMKRTTLDSPLGKLELSGCEQGLHRIIFLGKGTSAADAVEVPAPAAVLGGPEPLMQATAWLNAYFHQPEAIEEFPVPALHHPVFQQESFTRQVLWKLLKVVKFGEVISYSHLAALAGNPAATAAVKTALSGNPVPILIPCHRVVQGDLDVGGYEGGLAVKEWLLAHEGHRLGKPGLG][SGGGGSGGGGSGGGGS][QETELSVSAELVPTSSWNISSELNKDSYLTLDEPMNNITTSLGQTAELHCKVSGNPPPTIRWFKNDAPVVQEPRRLSFRSTIYGSRLRIRNLDTTDTGYFQCVATNGKEVVSSTGVLFVKFGPPPTASPGYSDEYEEDGFCQPYRGIACARFIGNRTVYMESLHMQGEIENQITAAFTMIGTSSHLSDKCSQFAIPSLCHYAFPYCDETSSVPKPRDLCRDECEILENVLCQTEYIFARSNPMILMRLKLPNCEDLPQPESPEAANCIRIGIPMADPINKNHKCYNSTGVDYRGTVSVTKSGRQCQPWNSQYPHTHTFTALRFPELNGGHSYCRNPGNQKEAPWCFTLDENFKSDLCDIPACDSKDSKEKNKMEILYILVPSVAIPLAIALLFFFICVCRNNQKSSSAPVQRQPKHVRGQNVEMSMLNAYKPKSKAKELPLSAVRFMEELGECAFGKIYKGHLYLPGMDHAQLVAIKTLKDYNNPQQWTEFQQEASLMAELHHPNIVCLLGAVTQEQPVCMLFEYINQGDLHEFLIMRSPHSDVGCSSDEDGTVKSSLDHGDFLHIAIQIAAGMEYLSSHFFVHKDLAARNILIGEQLHVKISDLGLSREIYSADYYRVQSKSLLPIRWMPPEAIMYGKFSSDSDIWSFGVVLWEIFSFGLQPYYGFSNQEVIEMVRKRQLLPCSEDCPPRMYSLMTECWNEIPSRRPRFKDIHVRLRSWEGLSSHTSSTTPSGGNATTQTTSLSASPVSNLSNPRYPNYMFPSQGITPQGQIAGFIGPPIPQNQRFIPINGYPIPPGYAAFPAAHYQPTGPPRVIQHCPPPKSRSPSSASGSTSTGHVTSLPSSGSNQEANIPLLPHMSIPNHPGGMGITVFGNKSQKPYKIDSKQASLLGDANIHGHTESMISAEL][SGGGGSGGGGSGGGGS][MVSKGEELFTGVVPILVELDGDVNGHKFSVSGEGEGDATYGKLTLKFICTTGKLPVPWPTLVTTLTYGVQCFSRYPDHMKQHDFFKSAMPEGYVQERTIFFKDDGNYKTRAEVKFEGDTLVNRIELKGIDFKEDGNILGHKLEYNYNSHNVYIMADKQKNGIKVNFKIRHNIEDGSVQLADHYQQNTPIGDGPVLLPDNHYLSTQSALSKDPNEKRDHMVLLEFVTAAGITLGMDELYK][HHHHHHHHHH] |

**Table S2:** Annotated plasmid sequence of the customized pCAG-ROR1-GFP construct from VectorBuilder.

| **ROR1 Targeting Sequence + SNAP-tag** |
| --- |
| ATGCACCGGCCGCGCCGCCGCGGGACGCGCCCGCCGCTCCTGGCGCTGCTGGCCGCGCTGCTGCTGGCCGCACGCGGGGCTGCTGCCGACAAAGACTGCGAAATGAAGCGCACCACCCTGGATAGCCCTCTGGGCAAGCTGGAACTGTCTGGGTGCGAACAGGGCCTGCACCGTATCATCTTCCTGGGCAAAGGAACATCTGCCGCCGACGCCGTGGAAGTGCCTGCCCCAGCCGCCGTGCTGGGCGGACCAGAGCCACTGATGCAGGCCACCGCCTGGCTCAACGCCTACTTTCACCAGCCTGAGGCCATCGAGGAGTTCCCTGTGCCAGCCCTGCACCACCCAGTGTTCCAGCAGGAGAGCTTTACCCGCCAGGTGCTGTGGAAACTGCTGAAAGTGGTGAAGTTCGGAGAGGTCATCAGCTACAGCCACCTGGCCGCCCTGGCCGGCAATCCCGCCGCCACCGCCGCCGTGAAAACCGCCCTGAGCGGAAATCCCGTGCCCATTCTGATCCCCTGCCACCGGGTGGTGCAGGGCGACCTGGACGTGGGGGGCTACGAGGGCGGGCTCGCCGTGAAAGAGTGGCTGCTGGCCCACGAGGGCCACAGACTGGGCAAGCCTGGGCTGGGT |
| **N-terminal Linker** |
| TCTGGTGGCGGAGGCTCGGGCGGAGGTGGGTCGGGTGGCGGCGGATCA |
| **ROR1 Protein Sequence** |
| CAAGAAACAGAGCTGTCAGTCAGTGCTGAATTAGTGCCTACCTCATCATGGAACATCTCAAGTGAACTCAACAAAGATTCTTACCTGACCCTCGATGAACCAATGAATAACATCACCACGTCTCTGGGCCAGACAGCAGAACTGCACTGCAAAGTCTCTGGGAATCCACCTCCCACCATCCGCTGGTTCAAAAATGATGCTCCTGTGGTCCAGGAGCCCCGGAGGCTCTCCTTTCGGTCCACCATCTATGGCTCTCGGCTGCGGATTAGAAACCTCGACACCACAGACACAGGCTACTTCCAGTGCGTGGCAACAAACGGCAAGGAGGTGGTTTCTTCCACTGGAGTCTTGTTTGTCAAGTTTGGCCCCCCTCCCACTGCAAGTCCAGGATACTCAGATGAGTATGAAGAAGATGGATTCTGTCAGCCATACAGAGGGATTGCATGTGCAAGATTTATTGGCAACCGCACCGTCTATATGGAGTCTTTGCACATGCAAGGGGAAATAGAAAATCAGATCACAGCTGCCTTCACTATGATTGGCACTTCCAGTCACTTATCTGATAAGTGTTCTCAGTTCGCCATTCCTTCCCTGTGCCACTATGCCTTCCCGTACTGCGATGAAACTTCATCCGTCCCAAAGCCCCGTGACTTGTGTCGCGATGAATGTGAAATCCTGGAGAATGTCCTGTGTCAAACAGAGTACATTTTTGCAAGATCAAATCCCATGATTCTGATGAGGCTGAAACTGCCAAACTGTGAAGATCTCCCCCAGCCAGAGAGCCCAGAAGCTGCGAACTGTATCCGGATTGGAATTCCCATGGCAGATCCTATAAATAAAAATCACAAGTGTTATAACAGCACAGGTGTGGACTACCGGGGGACCGTCAGTGTGACCAAATCAGGGCGCCAGTGCCAGCCATGGAATTCCCAGTATCCCCACACACACACTTTCACCGCCCTTCGTTTCCCAGAGCTGAATGGAGGCCATTCCTACTGCCGCAACCCAGGGAATCAAAAGGAAGCTCCCTGGTGCTTCACCTTGGATGAAAACTTTAAGTCTGATCTGTGTGACATCCCAGCGTGCGATTCAAAGGATTCCAAGGAGAAGAATAAAATGGAAATCCTGTACATACTAGTGCCAAGTGTGGCCATTCCCCTGGCCATTGCTTTACTCTTCTTCTTCATTTGCGTCTGTCGGAATAACCAGAAGTCATCGTCGGCACCAGTCCAGAGGCAACCAAAACACGTCAGAGGTCAAAATGTAGAGATGTCAATGCTGAATGCATATAAACCCAAGAGCAAGGCTAAAGAGCTACCTCTTTCTGCTGTACGCTTTATGGAAGAATTGGGTGAGTGTGCCTTTGGAAAAATCTATAAAGGCCATCTCTATCTCCCAGGCATGGACCATGCTCAGCTGGTTGCTATCAAGACCTTGAAAGACTATAACAACCCCCAGCAATGGACGGAATTTCAACAAGAAGCCTCCCTAATGGCAGAACTGCACCACCCCAATATTGTCTGCCTTCTAGGTGCCGTCACTCAGGAACAACCTGTGTGCATGCTTTTTGAGTATATTAATCAGGGGGATCTCCATGAGTTCCTCATCATGAGATCCCCACACTCTGATGTTGGCTGCAGCAGTGATGAAGATGGGACTGTGAAATCCAGCCTGGACCACGGAGATTTTCTGCACATTGCAATTCAGATTGCAGCTGGCATGGAATACCTGTCTAGTCACTTCTTTGTCCACAAGGACCTTGCAGCTCGCAATATTTTAATCGGAGAGCAACTTCATGTAAAGATTTCAGACTTGGGGCTTTCCAGAGAAATTTACTCCGCTGATTACTACAGGGTCCAGAGTAAGTCCTTGCTGCCCATTCGCTGGATGCCCCCTGAAGCCATCATGTATGGCAAATTCTCTTCTGATTCAGATATCTGGTCCTTTGGGGTTGTCTTGTGGGAGATTTTCAGTTTTGGACTCCAGCCATATTATGGATTCAGTAACCAGGAAGTGATTGAGATGGTGAGAAAACGGCAGCTCTTACCATGCTCTGAAGACTGCCCACCCAGAATGTACAGCCTCATGACAGAGTGCTGGAATGAGATTCCTTCTAGGAGACCAAGATTTAAAGATATTCACGTCCGGCTTCGGTCCTGGGAGGGACTCTCAAGTCACACAAGCTCTACTACTCCTTCAGGGGGAAATGCCACCACACAGACAACCTCCCTCAGTGCCAGCCCAGTGAGTAATCTCAGTAACCCCAGATATCCTAATTACATGTTCCCGAGCCAGGGTATTACACCACAGGGCCAGATTGCTGGTTTCATTGGCCCGCCAATACCTCAGAACCAGCGATTCATTCCCATCAATGGATACCCAATACCTCCTGGATATGCAGCGTTTCCAGCTGCCCACTACCAGCCAACAGGTCCTCCCAGAGTGATTCAGCACTGCCCACCTCCCAAGAGTCGGTCCCCAAGCAGTGCCAGTGGGTCGACTAGCACTGGCCATGTGACTAGCTTGCCCTCATCAGGATCCAATCAGGAAGCAAATATTCCTTTACTACCACACATGTCAATTCCAAATCATCCTGGTGGAATGGGTATCACCGTTTTTGGCAACAAATCTCAAAAACCCTACAAAATTGACTCAAAGCAAGCATCTTTACTAGGAGACGCCAATATTCATGGACACACCGAATCTATGATTTCTGCAGAACTG |
| **C-terminal Linker** |
| TCTGGTGGCGGAGGCTCGGGCGGAGGTGGGTCGGGTGGCGGCGGATCA |
| **EGFP + x10 His-tag** |
| ATGGTGAGCAAGGGCGAGGAGCTGTTCACCGGGGTGGTGCCCATCCTGGTCGAGCTGGACGGCGACGTAAACGGCCACAAGTTCAGCGTGTCCGGCGAGGGCGAGGGCGATGCCACCTACGGCAAGCTGACCCTGAAGTTCATCTGCACCACCGGCAAGCTGCCCGTGCCCTGGCCCACCCTCGTGACCACCCTGACCTACGGCGTGCAGTGCTTCAGCCGCTACCCCGACCACATGAAGCAGCACGACTTCTTCAAGTCCGCCATGCCCGAAGGCTACGTCCAGGAGCGCACCATCTTCTTCAAGGACGACGGCAACTACAAGACCCGCGCCGAGGTGAAGTTCGAGGGCGACACCCTGGTGAACCGCATCGAGCTGAAGGGCATCGACTTCAAGGAGGACGGCAACATCCTGGGGCACAAGCTGGAGTACAACTACAACAGCCACAACGTCTATATCATGGCCGACAAGCAGAAGAACGGCATCAAGGTGAACTTCAAGATCCGCCACAACATCGAGGACGGCAGCGTGCAGCTCGCCGACCACTACCAGCAGAACACCCCCATCGGCGACGGCCCCGTGCTGCTGCCCGACAACCACTACCTGAGCACCCAGTCCGCCCTGAGCAAAGACCCCAACGAGAAGCGCGATCACATGGTCCTGCTGGAGTTCGTGACCGCCGCCGGGATCACTCTCGGCATGGACGAGCTGTACAAGCACCACCACCATCACCATCATCACCACCACTAA |
